# Gaps in TB-related knowledge and practices: An assessment of health care seeking behavior among adults with HIV and caregivers of paediatric patients with presumptive TB symptoms in Manhiça district, southern Mozambique

**DOI:** 10.1371/journal.pgph.0004734

**Published:** 2025-08-18

**Authors:** Agostinho Viana Lima, Hermínio Cossa, Hélder Djive, Otília Cossa, Miguel Cumbe, Sozinho Acácio, Babongile Nkala, Joachim Nsubuga Kikoyo, Lucia Carratala-Castro, Joanna Ehrlich, Sabine Hermans, Alexander Kay, Willy Ssengooba, Anna Mandalakas, Christoph Lange, Cristina Enguita-Fernàndez, Khátia Munguambe, Alberto L. Garcia-Basteiro, on behalf of the Stool4TB Global Partnership

**Affiliations:** 1 Centro de Investigação em Saúde da Manhiça (CISM), Manhiça, Mozambique; 2 College of Medicine and Texas Children’s Hospital, Baylor, Eswatini; 3 Department of Medical Microbiology, and Makerere University Lung Institute, Makerere University, Kampala, Uganda; 4 Barcelona Institute for Global Health (ISGlobal), Barcelona, Spain; 5 Department of Global Health, Amsterdam UMC, location University of Amsterdam, Amsterdam Institute for Global Health and Development, Amsterdam, Netherlands; 6 Department of Infectious Diseases, Amsterdam UMC, location University of Amsterdam, Centre of Tropical Medicine and Travel Medicine, Amsterdam, The Netherlands; 7 Baylor College of Medicine and Texas Children’s Hospital, Houston, Texas, United State of America; 8 Research Center Borstel, Leibniz Lung Center, Borstel, Germany; 9 German Center for Infection Research (DZIF), Hamburg-Lübeck-Borstel-Riems, Borstel, Germany; 10 Respiratory Medicine & International Health, University of Lübeck, Lübeck, Germany; 11 Faculty of Medicine, Eduardo Mondlane University, Maputo, Mozambique; Human Sciences Research Council, SOUTH AFRICA

## Abstract

Although tuberculosis is a preventable and treatable disease, its management has been challenging for tuberculosis control and prevention programs in low- and middle- income countries such as Mozambique. We assessed the TB knowledge and healthcare-seeking behaviors among adults with HIV and caregivers of paediatric patients with symptoms of TB. The study was conducted between February and October 2023 at Manhiça District Hospital. A total of 60 interviews were conducted with people with HIV and caregivers of paediatric patients showing symptoms of TB. The interviews were transcribed, coded using an excel matrix, and analyzed using a content analysis approach. Half of the participants recognized airborne transmission through coughing as the main mode of TB transmission, while others were unsure or linked TB to sociocultural beliefs. Coughing was identified by most as the main symptom, with some also mentioning chest pain, bleeding, fatigue or weakness, weight loss, fever and night sweats. Many respondents believed that avoiding sharing utensils was the main way of preventing TB. Respecting the respondents’ reasons and time taken to seek health care, our findings revealed that most participants had experienced the persistent coughing for more than 3 weeks. Some sought medical care, but did not see any improvement, while others chose to wait for their next scheduled doctor visit, hoping to address their symptoms. Our results showed that delays in seeking care were common among participants with TB symptoms, reflecting limited awareness of the disease. Factors such as waiting for the next scheduled doctor’s visit, misinterpreting symptoms, and misconceptions about TB may have contributed to these delays. To address this, raising awareness about TB transmission, symptoms and prevention, dispelling myths through health education, and improving TB symptom follow-up through a coordinated approach across various patient entry points are essential.

## Background

Tuberculosis (TB) remains the world’s leading cause of death from a single infectious agent despite being a preventable and treatable disease [1–3]. In 2023 alone, approximately 10.8 million people fell ill with TB and 8.2 million people were newly diagnosed with the disease, of whom 12% were children and young adolescents [3]. In the same year, TB accounted for 1.25 million deaths, of which 1.09 million were HIV-negative and 161,000 were people with HIV (PWH) [3]. Furthermore, thirty high TB burden countries, including Mozambique, bear 87% of the TB cases worldwide [3–5]. For instance, Mozambique, a country located in southern Africa, ranked among the 11 countries with the highest burden of TB, TB/HIV, and multidrug-resistant TB (MDR-TB) in 2023, with an estimated incidence of 300–499 new cases per 100,000 population [3,5,6].

Although the world has made progress in reducing the number of deaths caused by TB by 23% between 2015 and 2023, this is still far from achieving the WHO End TB Strategy milestone of a 75% reduction by 2025 [3]. Numerous studies have highlighted that the delay in diagnosis and treatment of TB is still a major challenge of tuberculosis control and prevention programs in low- and middle-income countries such as Mozambique [7–11]. In these contexts, the management of TB in children and adult PWH is further hampered by several factors. On one hand, the local misconceptions about TB transmission, the presence of non-specific signs and symptoms, and the slow clinical progression within these groups complicate timely diagnosis, and treatment [10,12–16]. For instance, previous studies conducted in the same study setting have shown that limited knowledge of TB, particularly its symptoms, diagnosis and treatment, among both caregivers of paediatric patients and health professionals has negatively affected patient outcomes and undermined national efforts to control the disease [16,17]. On the other hand, a study conducted in Uganda has revealed that health system factors such as lack of training, low staff motivation, and high workload, along with contextual factors including time and cost borne by patients to seek and complete TB evaluation, poor health literacy, and stigma against patients contribute to missed opportunities in TB investigation at public health facilities [18].

Moreover, the diagnosis of pulmonary TB typically relies on sputum samples, which are often paucibacillary and challenging to collect from PWH and children [19]. Limited sputum production and reduced sensitivity of diagnostic tools in detecting *M tuberculosis* in paucibacillary sputum limit its utility [20,21]. The objective of this study was to explore the knowledge about TB and healthcare-seeking behaviors, particularly among PWH and caregivers of paediatric patients to identify gaps related to these populations’ behaviors.

## Materials and methods

### Study design and setting

**Fig 1:**
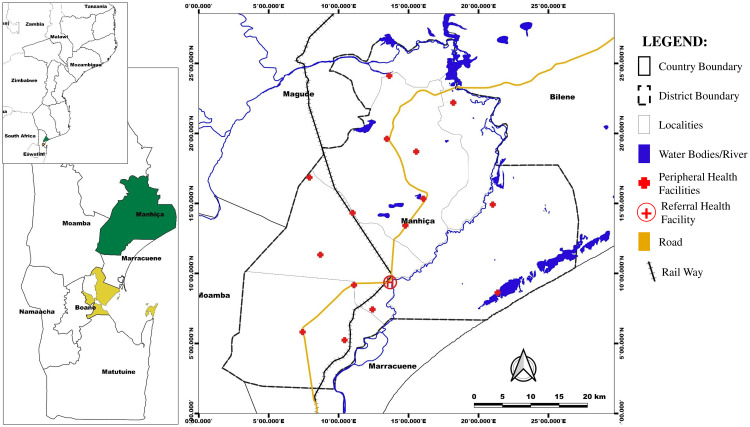
Map of the Manhiça District. Source: created by the Author using QGIS software (version 3.2.3-Bonn), country boundaries admin 3 level, and line features (i.e., waterways, roads and railways lines) *shapefile* are from The Humanitarian Data Exchange (https://data.humdata.org/). The direct links to each *shapefiles* data are as follow - country admin 3 level (direct link), road line *shapefiles* (direct link), railway line *shapefiles* (direct link), waterways *shapefiles* (direct link). Health facilities’ GPS data was acquired from the Manhiça Health and Demographic Surveillance System (HDSS) data of the Manhiça Health Research Centre (CISM) [30].

This generic interpretative qualitative study was embedded in a multi-country project evaluating a novel stool-based qPCR diagnostic assay (Stool4TB) [19]. It was conducted in Manhiça, a rural district in Southern Mozambique, located approximately 90 km from Maputo city, the country’s capital (Fig 1). Manhiça covers an area of 2360 square kilometers, and is home to around 49,000 inhabited households with a total population of approximately 266,435 people [22–24]. The Manhiça District has two hospitals, and it is estimated that the prevalence of HIV infection among adults in the district is 35%, with an incidence of HIV among adolescent girls and young women ranging from 0.3% to 1% [22,25–27]. In 2012, the TB case notification rate was 552 per 100,000, with significantly higher rates observed among people with HIV [28]. A substantial proportion of the Manhiça population works in commerce, fishery, and subsistence farming, with some working as laborers in two sugar cane plantations and refineries, and others employed by small agricultural farms [29,30].

### Study respondents, recruitment and data collection

A total of 60 participants were recruited between 9^th^ February 2022 and 9^th^ March 2023 at Manhiça District Hospital, including 20 PWH and 40 caregivers of paediatric patients with symptoms of TB. Participants were recruited at the exit of the consultation room, using a combination of purposive and convenience sampling methods [31,32]. The purposive sampling technique, also called judgment sampling, is a type of non-probability or non-random sampling in which researchers deliberately select participants based on specific characteristics they possess [32]. On the other hand, the convenience sampling is another non-probability approach where participants are selected from the target population based on their ease of access [31,32]. The selection criteria included: adult PWH aged ≥ 18 years of age or caregivers responsible for a child aged ≤ 8 years who are seeking care at the main study health facility (Manhiça District Hospital) with presumptive TB, enrolled in the Stool4TB clinical study, and willing to participate in the Socio-Behavioral Study (SBS).

Data were collected through exit-interviews (EIs) (S1 Text), which consisted of open-ended questions lasting between 30 and 45 minutes. The interview guide was designed to explore the opinion of PWH and caregivers about presumptive TB status and health-seeking behavior.

Two trained social sciences research assistants (HD and OC) collected the data. Both fieldworkers were fluent in Portuguese (the national language of Mozambique) and Xichangana (local language) and were trained in protocol procedures and qualitative data collection methods. The research assistants were supervised by a study coordinator (AL), who reported directly to the local Principal Investigator (KM). Interviews were conducted in either Xichangana or Portuguese, depending on participant preferences, and audio-recorded with their consent.

### Data management and analysis

#### Transcriptions and storage.

The interview recordings were transcribed verbatim and translated simultaneously from Xichangana to Portuguese by the research assistants (HD and OC). Subsequently, selected excerpts were later translated from Portuguese to English by a professional translator. During this translation process, repeated words, incomplete sentences, and expressions of hesitation were removed to improve readability, while missing or implied words were inserted in brackets to preserve the intended meaning and ensure clarity. To ensure data safety and privacy, all recordings, transcripts, and summaries were stored at the Manhiça Health Research Center on a password-secured data server, ensuring that only study members had access to the data.

#### Data quality control.

To ensure data quality, the study team proceeded with revision of both recording and transcripts throughout the course of the study [33,34]. First, the study coordinator reviewed each recording by listening to its content to evaluate if all questions were made as follows or asked in the interview guide, checked the quality of the responses, as well as the opportunities for prompts and probes. Together with the principal investigator, the coordinator discussed the findings of the data quality control to design better corrective actions to improve the quality of the data. Next, the corrective actions were regularly discussed with the research assistants to ensure their implementation.

As with the recordings, the study team ensured quality control of the transcripts. This process involved listening to the audio recordings and comparing them with the corresponding transcripts. Any findings or inconsistencies were recorded in the same document through amendments. The file, including the findings, was then returned to the assistants/transcribers for the necessary corrections. In the event of a disagreement, the supervisor and transcriber discussed the discrepancies until they reached a consensus. Finally, the corrected transcript was archived for the final data analysis.

#### Database creation.

For this research, the study coordinator accessed the stored data on July 4th, 2024, to develop the writing plan. During this process, two separate databases were created and analyzed based on the study questions. The first one was a qualitative database that explored the participants’ accounts of their experiences, perceptions, knowledge, and attitudes towards TB. The second database was for quantitative analysis that explored the frequency of the responses and their tendencies among the target groups.

#### Qualitative analysis.

The transcripts were summarized using the interview guide in a Microsoft Excel matrix, with the participants’ ID in rows and the themes, topics and nodes in columns. The themes corresponded to predetermined questions that guided the data collection process, such as participants’ knowledge of TB, reasons for seeking health care, time taken to seek health care, suspicions of illness and initial actions to relieve symptoms.

**Fig 2:**
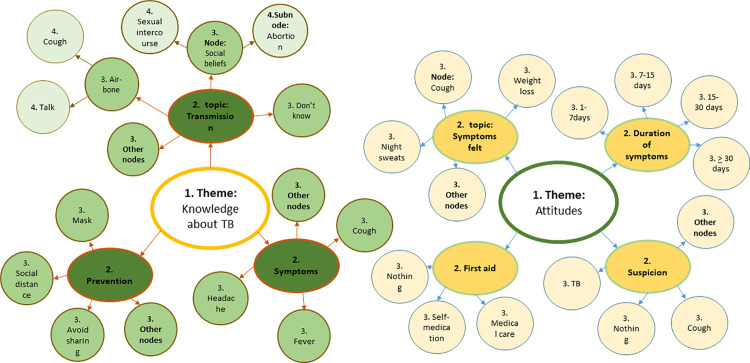
Code map.

For each theme, specific topics were identified, such as “knowledge of TB transmission”, and within those topics, nodes were generated, for example, “airborne transmission through cough”. As the data was analyzed through content analysis [35], new nodes emerged from thematic analysis [36] and were integrated into the existing topics and themes. The matrix was thus structured to reflect this hierarchy: each theme addressed a specific predetermined question, each topic was linked to a theme, and each node was associated with a corresponding topic (Fig 2).

Participants’ excerpts were then entered into the cells of each node. Finally, a summary of key insights was created for each topic and related nodes, with illustrations using in-text citations and tables of reporting frequencies. The presentation of findings complied with the Consolidated Criteria for Reporting Qualitative Research (COREQ) guidelines [37].

#### Quantitative analysis.

We developed a quantitative database using Microsoft Excel (Microsoft Office Standard 2016, v16.0.54, 64 bit) by transforming the qualitative database, where we recoded nodes into variables (e.g., themes or concepts) and assigned “yes” or “no” values based on the content of participants’ responses (excerpts) for each node. For instance, if a participant mentioned ‘coughing’ as a transmission mechanism, the variable ‘coughing’ was assigned the value “yes.” The numerical variable ‘age’ was converted into a categorical variable with three categories: young adults (18–35), adults (36–65), and the elderly (66+). The quantitative database was then imported into R software (version 4.3.1, dated 16.06.23). Using packages such as GTSummary, tidyverse, and Dplyr, a descriptive analysis was performed by calculating the frequency of the responses for each variable. The results were then presented in a tabulated form.

#### Triangulation.

Using both the qualitative and quantitative databases, we conducted a content analysis to summarize the key insights for each theme [35,38]. The data were presented in a way that highlighted the percentage of respondents, illustrating trends in their responses, while quotes were included to provide a deeper understanding of the meaning emerging from participants’ answers. Finally, these insights and interpretations were cross-referenced with findings from similar studies to enhance the validity of the results.

### Ethical approval

This study was conducted according to the protocol, the Declaration of Helsinki, as well as other locally relevant regulations. Administrative approval was first obtained from the Manhiça District Government (Ref. 1892/GDM/GA/115/2021), followed by ethical approval from the Institutional Review Board of the Manhiça Health Research Centre (Ref. CISM-014–2021) in Mozambique. Written informed consent was obtained from all participants before any data collection procedures. Thumbprints of those who were illiterate were taken in the presence of an impartial witness who signed the consent form as well, ensuring that participants were informed and willingly participating. In addition, all interviews were conducted taking into account the linguistic comfort of each respondent. Following the interviews, all recordings and field notes were anonymized using identifiers such as “Male Caregiver 01” or “Female Adult Patient 01”. However, only the principal investigator, study coordinator, and research assistants had access to information that could identify individual participants during and after data collection.

## Results

### Socio-demographic characteristics

In this study, we interviewed 20 adult patients and 40 caregivers of paediatric patients. The overall mean age of all participants was 38 years, with adult patients being generally older than caregivers, who had a mean age of 32 years. The majority of the participants were female caregivers of paediatric patients who had no contact with a TB case. Nonetheless, there were differences between the groups, with caregivers more likely to be married, compared to adult patients. Despite this, both groups had a low level of education, with adult patients showing a tendency towards lower literacy; the majority of caregivers had attended at least some form of schooling, compared to adult patients who had little or no formal education. Farming was the primary source of income for the majority of our participants (Table 1).

**Table 1:** Sociodemographic characteristics.

Characteristics	Adult patients Total (n = 20)	Caregivers Total (n = 40)
Age Group		
Young adults (18–35)	4 (6.7%)	26 (43.0%)
Adults (36–65)	14 (23.0%)	14 (23.0%)
Elderly (66+)	2 (3.0%)	0 (0.0%)
Mean: 40, SD [14]	34, SD [10]	50, SD [14]
Gender		
Female	11 (18.0%)	39 (64.0%)
Male	9 (15.0%)	1 (1.7%)
TB Status		
Cases	0 (0.0%)	0 (0.0%)
Contacts	0 (0.0%)	13 (%)
Religion		
Christian	20 (33.0%)	38 (63.0%)
Muslim	0 (0.0%)	2 (3.0%)
Marital status		
Single	13 (21.7%)	10 (16.7%)
Married	6 (10.0%)	25 (41.7%)
Widowed	1 (1.7%)	5 (8.0%)
Education Level		
Illiterate	8 (13.0%)	3 (5.0%)
Incomplete Primary level	6 (10.0%)	15 (25.0%)
Primary level completed	3 (5.0%)	3 (5.0%)
Incomplete Secondary level	3 (5.0%)	8 (13.0%)
Secondary level completed	0 (0.0%)	10 (16.7%)
High school	0 (0.0%)	1 (1.7%)
Sector		
Public	0 (0.0%)	4 (6.7%)
Private/NGO	0 (0.0%)	1 (1.7%)
Informal	20 (33%)	35 (87.5%)

### Knowledge about tuberculosis (transmission, symptoms and prevention modes)

#### Modes of transmission.

Half of the respondents, particularly caregivers, identified air-bone transmission through coughing as the main route of TB transmission. Only a few of them mentioned saliva droplets produced when speaking as another potential route of transmission (Table 2). For instance, Participant 11, an adult female patient, said “[*Tuberculosis is transmitted] when someone coughs* […] *and the air comes to you.* [*So*] *there you can catch the disease*”.

**Table 2:** Knowledge of TB transmission forms.

Question: How can someone catch TB?		
Answers	Number of respondents and frequency n/N (100%)	Quotes
TB is transmitted through the air by cough	30/60 (50%)	“[TB is transmitted] when someone coughs [...] and the air comes to you, then you can catch the disease” (Female Adult Patient 11)
Don’t know	13/60 (22%)	“That (how TB is transmitted) (...), I can’t even know how [to] catch it” (Female Caregiver 03)
Non-compliance with traditional rules(11): Engaging in sexual relations with someone, or using something belonging to a family that has not yet undergone a purification ritual following the death of a relative.	11/60 (18%)	“This disease is contracted [...] when you take the clothes of someone who has lost someone, even if they are your brother or sister. If you don’t follow the rituals and take them home, you can catch the disease” (Male Adult Patient 25) “[You get TB] from having sex, it’s a lot. Sometimes if someone has died in the house and they don’t follow the recommendations (rituals), one of the people can contract the disease” (Female Adult Patient 22)
Sharing objects or space with someone with TB.	5/60 (8%)	“[You catch it] [...] by using objects like, for example, [...] a blade that someone has used who has tuberculosis [...] or having used a glass that someone with tuberculosis has used. [There are various ways [of transmitting it]” (Female Caregiver 11).
Getting in touch with droplets of saliva from someone with TB.	6/60 (10%)	“They explained to me that talking can transmit that, if that comes out, if saliva comes out, he playing with his friends, he can transmit this tuberculosis disease” (Female Caregiver 11).
Having sex with a woman who has had an abortion	2/60 (3%)	“It’s transmissible when you have sex with a woman who has had an abortion. And you come in (I mean, you have sex with her), without telling her” (Male Adult Patient 08).
Getting in touch with dust	1/60 (2%)	“[…] my husband got sick [...] because he worked in the fields. [...] So, we think he caught it there because of that dust, from those chickens. When it’s chickens, I don’t know what they use, sawdust, bran, lots of things, right, and then he started coughing. We saw that the disease was getting worse and we took him to the hospital, [where] he was diagnosed with tuberculosis” (Female Caregiver 15).
Smoke	1/60 (2%)	“Tuberculosis is a disease that appears in people who smoke cigarettes. It rots the lungs […]” (Male Adult Patient 16).

Nonetheless, many participants, particularly adult patients, demonstrated a limited understanding of TB transmission. Some admitted they did not know how TB is transmitted, while others expressed misconceptions rooted in sociocultural beliefs. Common misconceptions included the idea that TB could be transmitted by touching objects or through sexual contact with someone whose family had not carried out traditional purification rituals following a relative’s death, or with a woman who had undergone an abortion.

One female caregiver expressed her uncertainty, saying, “*It (the way TB is transmitted) (…), I can’t even know if it caught*” (Female Caregiver 03).

Further illustrating the sociocultural beliefs, a male adult patient shared, “*This disease we contract […] when you take (use) clothes from someone whose relative has passed away, even if it’s your brother (who died)*. *If you don’t follow the rituals and take [the clothes] to your home, you can catch the disease*”.

Additionally, some participants associated it with sharing objects or spaces with TB patients, or exposure to dust or smoke, further highlighting the diverse sociocultural interpretations surrounding the disease.

#### TB symptoms.

Regarding the symptoms of TB (Table 3), most respondents identified cough as the main symptom of TB, with only a few of them associating it with other symptoms such as cough with chest pain, bleeding, fatigue or weakness, weight loss, fever and night sweats. A male adult patient elaborated, “*Yeah, I…when somebody has TB has the problem of coughing and then bleeding or coughing and not producing any dirt (sputum) […]*”, highlighting a common understanding of cough and related complications.

**Table 3:** Knowledge of symptoms of tuberculosis.

Question: How can you recognize that someone has TB?			
Answers		Number of respondents and frequency n/N (100%)	Quotes
Symptoms of unwellness	Cough with chest pain* or blood**	34/60 (57%) (2*, 3**)	“Yeah, […] when someone has tuberculosis they have problems of [...] coughing and then getting that blood out or coughing very badly and not getting dirt out (sputum) […]” (Male Adult Patient 09). “[he has] a cough [and] chest pain. I can’t stand it. I know, because you see that this person is sick, but they cough and feel chest pain, and they say it’s tuberculosis” (Female Adult Patient 20).
	Fatigue or weakness	13/60 (22%)	“[…] I’ve heard of it. You lose weight, you get weak and almost lose your strength, you don’t feel like eating, you have no appetite for food” (Female Adult Patient 26).
	Fever, headache or vomit	7/60 (12%)	“Fever [and] vomiting, [...] that’s more or less what I know” (Male Caregiver 06). “The symptoms of this disease are fever, headache […]” (Female Adult Patient 21).
	lack of appetite	7/60 (12%)	“I can’t lie to you, but they say that they lack appetite, you are tired, it hurts […] when you talk […], something like that. I have never been ill” (Female Caregiver 10).
	Night sweats	6/60 (10%)	“[…] night sweat, cough for two weeks, fever […]”” (Female Caregiver 26).
	Stress	1/60 (2%)	“Sometimes someone feels head[ache], cough, gets thin and their hair gets a bit…you see, and gets nervous” (Male Adult Patient 09).
Physical changes	Weight loss(18)	18/60 (30%)	“[…] the people I have seen, some lose their weight, they lose even their ability to work, you see!” (Male Adult Patient 23).
	Experience hair loss or changes in capillary characteristics	4/60 (7%)	“[…] when a person had tuberculosis, we could see that they were breaking up, they were losing weight, their hair was [flat] [...] like a white person. That’s how we realized that this person had tuberculosis” (Male Adult Patient 14). “People who have this disease, you can see by their appearance, [...] [that] the disease has already consumed them a bit (they’ve lost weight), their hair is scattered and they’re running out, they look unpleasant. Then you say, No, this person is breaking up. [...] but [...] I can see it in his body, in his appearance [...] and hair” (Male Adult Patient 18).
	Develop darker skin	1/60 (1.7%)	“[…] coughing a lot [...] unlike a cold, [...] coughing all the time, [...] getting dark, losing weight [...]. I don’t know of any other symptoms” (Female Caregiver 11).
Don’t know		10/60 (16.7%	“Um, that’s a bit difficult. I wouldn’t [be able to say what the symptoms of TB would be], as perhaps I’ve never lived with this person (someone with TB), it’s difficult” (Female Caregiver 11).

Another participant expanded this view, stating, “*Sometimes a person feels headache, coughs, loses weight and their hair turns one and one (start losing hair)…can you see, and they get nervous*” (Male Adult Patient 09), showing a broader but less conventional perception of symptoms.

This view was prevalent among some respondents who mentioned non-specific signs such as hair loss, stress, and skin darkening as symptoms or admitted not knowing TB symptoms. Reflecting this lack of awareness, a female Caregiver shared, “*Hum, it is a bit difficult. No [I couldn’t say what the symptoms of TB were], as I have never seen a person (with TB), it is difficult*” (Female Caregiver 11). These varied responses underline the gaps in understanding and the need for targeted education on TB symptoms.

#### TB prevention modes.

When asked how to prevent TB, several participants correctly mentioned TB preventive methods such as social distancing, seeking and complying with medical recommendations, wearing masks, adopting proper cough etiquette, keeping living spaces well-ventilated, and avoiding crowded places (Table 4). A female Caregiver highlighted these preventive actions, stating, “*We must keep wearing masks. A person must try to know their (health) status and keep the house windows open, clean it (the house)*” (Female Caregiver 27).

**Table 4:** Knowledge of ways to prevent tuberculosis.

Question: How can someone prevent her/himself from TB?		
Answers	Number of respondents and frequency n/N (100%)	Quotes
Avoid sharing objects and utensils with someone with symptoms of TB	26 (43%)	“Because I know I’m not well, I can’t exchange my can with my friend. Another thing I know you have to respect is having control over my spoon, nobody uses it. My plate not being used, being mine, but talking, smiling, living, being the same as before, um” (Male Adult Patient 14).
Keep social distance from with someone with symptoms of TB	23 (38%)	“The way to prevent this is for me to have my plates and spoons, because my plate is my plate and my spoon is mine. And when it comes to sleeping, distance yourself from the woman, because when you sleep next to her, there’s an exchange of breaths. [...] I, who feel the pain, can distance myself from her […]” (Male Adult Patient 16).
Seek for health care and comply with medical recommendation	18 (30%)	“To prevent this disease, we must go to the doctor for treatment” (Female Adult Patient 21). “[…] the best prevention is [...] to go to the hospital, [and] when they tell you this, you have to comply [with] what they’re telling you, [that] you’ll get out of it” (Male Adult Patient 14).
Use mask	12 (20%)	“Putting on a mask [is the] first thing. Wearing a mask. Care like this [...] children at school and even here at home will be a bit difficult, because she has to stay I don’t know inside, alone or with a mask on […]” (Female Adult Patient 26).
Don’t know	9 (15%)	“Prevention of this disease is (silence), [...] I don’t know how I can prevent it” (Male Adult Patient 19).
Comply with the cough etiquette	9 (15%)	“With a person with tuberculosis at home, the method of prevention is to get together with them, advise them to use their glass, a spoon, their plate and when they cough, don’t cough anywhere, get something or if they want to spit, they can take a cloth and close their mouth and spit, like this” (Female Adult Patient 21). “The person who has tuberculosis has to either close their mouth or wear a mask when coughing. What I know is that to follow the recommendations, you always have to take precautions. You either have to stay away from them (family members) for a while, or I have to wash the dishes I eat on my own. The plate and spoon have to be my things” (Male Adult Patient 18).
Keep individual and collective (house) hygiene	6 (10%)	“When you go to the bathroom, you should always have water to wash your hands with soap or ash when you feed them” (Female Caregiver 22).
Keep the house or the place ventilated	3 (5%)	“Hum, one of the things [is that] the room can’t be too stuffy, you have to [...] open the windows to let the sun in, to air it out” (Female Caregiver 19). “You have to wear a mask. You have to find out about your condition and leave the windows open, clean the house” (Female Caregiver 27).
Comply with traditional rules of purification	2 (3%)	“[...] in order not to catch [this] disease, most of us, I can mention my brother-in-law, lost his life because he didn’t follow the rituals after a death. There was like an argument between us, because I had two wives, I did the bath with my wives, we followed the recommended rituals and we got out of it (we prevented ourselves) […]” (Male Adult Patient 23).
Avoid crowding	2 (3%)	“Places where alcohol is consumed, you can sit here or there, that’s it. Then we can prevent the disease from contaminating us. And know that, in places where people are crowded, I have to stay away, when I realize that the air is going that way, you sit on the other side, they’re going to cough that way, and you stay on this side. You have to realize the direction of the air, don’t sit on this side, because everything they cough turns towards you” (Male Adult Patient 19).

However, some participants framed prevention in more personal or less conventional terms. For instance, a male adult patient shared, “*How to prevent (tuberculosis) is for me to have my plates and spoons […]*” (Male Adult Patient 16), reflecting an individual approach rooted in the perceived risk of sharing utensils. These insights illustrate both awareness of effective strategies and the persistence of personal or culturally informed methods, highlighting areas for strengthening TB prevention education.

On the other hand, several participants, particularly caregivers, demonstrated incorrect knowledge about TB prevention. They believed that avoiding sharing utensils or personal items with a TB patient was sufficient for prevention. A male adult patient articulated this perspective: “*Because I know that I don’t feel good, I can’t exchange my cup with my friend. Another thing I know that must be respected is taking control over my spoon; do not let anybody use it. My plate must be mine, but to talk, smile, live, and be the same one you used to be, hum*” (Male Adult Patient 14).

Additionally, a few participants admitted to not knowing how to prevent TB, while a couple of them recognized family purification rituals as a method of TB prevention, indicating significant gaps in awareness. This belief was captured by one participant, who stated, *“[…] to not catch [this] disease, most of us, I can talk about my brother-in-law, has died due to not following the rituals after a death (of somebody). It was like an argument between us (he and I), because I had had two wives, I did the bath (a ritual) with my wives, we followed the recommended rituals and we got out of this (we prevented ourselves from the TB) […]*” (Male Adult Patient 23).

These findings highlight the persistence of culturally informed misconceptions about TB prevention and the need for tailored health education to address these gaps.

### Health seeking behavior of TB symptomatic patients

#### Patients’ symptoms and reason for seeking health care.

Persistent cough was the primary symptom and the most cited reason for seeking-healthcare, across all participant groups (Table 5). For example, Participant 11, a male adult participant said “*What made me come to Hospital today it is cough. There are* […] *two weeks that I have been coughing. Hah, I thought it was [normal] cough and that it would pass but it doesn’t pass*”.

**Table 5:** Participants’ symptoms.

Question: What do you feel?		
Answers	Number of respondents and frequency n/N (100%)	Quotes
Cough	50 (83%)	“What made me come to the hospital today is that I’ve been coughing. I’ve been coughing for [...] two weeks. I thought it was a cough and that maybe it would go away, but nothing does” (Male Adult Patient 14).
Fever	8 (13%)	“Because of his illnesses, he has a cough and a fever” (Female Caregiver 08)
Antiretroviral pills collection as the main reason for seeking healthcare	7 (12%)	“Today I came to check the pills, I always take, but the rule says that when I come to take the pills I have to go to the school, where they tell us about the analysis of the medicines we take. I told [her] that I had a cough [since] May. [But] it’s been getting worse this month (June). Now I’ve been coughing up dirt (Male Adult Patient 23).
Weight loss	8 (13%)	“Yes, [...] he (the child) has a cough that doesn’t go away. [...] he had [...] a cough every two weeks. One week would pass, and the next week it would start again [...] he was already getting thin. So I got worried, [and] said, no, let’s see what’s happening to him. [That’s why [...] I went to the hospital so that I could do the tests to find out what he has” (Female Caregiver 08)
Night sweat	5 (8%)	“What brought me to the hospital today is that I don’t feel well, and I sweat a lot when I’m sleeping. I cough a bit [...] and when I’m sleeping, I sweat [...]. So I decided to go to the hospital, they (health workers) know what kind of illness it might be” (Male Adult Patient 18).
Chest pain	7 (12%)	“What brought me to the hospital was a chest problem, it was hurting a lot. When I cough, it looks like a real wound is [there] and dirt comes out [...]. That’s what led me to go to the hospital” (Female Adult Patient 13).
Headache	5 (8%)	“What made me come to the hospital today was [...] feeling cold, then [...] my ribs hurt, it felt like they had been pricked and stretched. So, when I slept [...], I couldn’t turn over, [...] my head hurt and [...] I said [...] I’m not well, I have to go to the hospital […]” (Male Adult Patient 15).
Weakness	3 (5%)	“[...] now I have chest problems that leave me without strength and energy, that I can’t even respond to. I stay still, someone else only sees my eyes blinking, while I feel like I’ve died” (Male Adult Patient 15).
Body pain (full body(3), feet(2) or rib(2))	7 (12%)	“[...] what I feel in my body is my foot and my headache. And my body always hurts around here (chest), as if something were eating me. Cough with nothing coming out. [...] my illness is just coughing and [...] a headache. [...] my head has been aching for a long time, my head is always aching... my body aches, but at night I have a fever and I sweat, I sweat until I drip” (Female Adult Patient 07).
Others*	15 (25%)	“She doesn’t feel anything. I brought her in because her grandmother is ill, and as she likes to carry her around, feed her, she may have passed it on to her.” (Female Caregiver 08)

Legend: *Wounds(2); vomit(1); throat swelling and irritation(2); breathing difficulties(2); lack of appetite(2); nothing (contact of a TB-positive patient)(1); stomach pain and diarrhea(1).

In addition, Participant 8, a female caregiver, explained that “[…] *he* (*the child*) *has a cough that doesn’t go away. It would go away in a week and start the following week.* […] [*and*] *he was getting thin (losing weight)* [*…*]. [*That’s*] *why I came to the hospital to be able to examine (him) to find out what he (the child) has*” (Female Caregiver 08).

TB-related symptoms such as fever, weight loss, night sweats, chest pain, headache, and weakness were often reported as secondary reasons for seeking health care. For instance, a male caregiver noted, “*Fever [and] vomiting, [...] that’s more or less what I know*” (Male Caregiver 06), while a female adult patient added, “*The symptoms of this disease is fever, headache […]*” (Female Adult Patient 21).

In contrast, some who were PWH mentioned collecting antiretroviral (ARVs) pills as their main reason for seeking health care, despite having experienced one or more TB-related symptoms. One male adult patient described his situation, stating, “*Here, at the hospital, I came to collect pills that I take every three months. [However] […] I feel […] always pain around here (chest), as if something were eating me. I cough, and nothing comes out. [...] my illness is just coughing and [...] a headache. [...] my head has been aching for a long time, my head is always aching... my body aches, but at night I have a fever and I sweat, I sweat until I drip*” (Male Adult Patient 07). This reflects a disconnection between recognizing symptoms and seeking care specifically for TB.

Of note, among the caregivers of paediatric patients, only three reported seeking health care for their children even when the children did not show any visible TB-related symptoms. This proactive behavior appeared to be influenced by health professionals’ instructions to bring their children to the health facility for medical exams. A female caregiver explained, “*[I have thought] that may be [the child] has tuberculosis because at home there is someone (their grandmother) who has it*” (Female Caregiver 27). Another caregiver elaborated, “*[the child] has nothing. I brought him/her because their grandmother is sick (she has TB), [and] as she likes to hold them, [and] feed [them], she might have passed on the disease*” (Female Caregiver 08).

These findings highlight diverse motivations for seeking health care, with some participants prioritizing other conditions over TB symptoms, while others followed preventive advice for potentially exposed children, underscoring gaps and opportunities for TB awareness and early intervention.

#### Attitudes towards symptoms (suspicions, time needed to seek health care, and action taken to cure or relieve the symptoms).

The majority of respondents either attributed their symptoms to no illness or conditions such as cough, asthma, HIV complications, malaria, ARV side effects, fatigue, COVID-19, or stomachache (Table 6). In contrast, some participants, especially those with a persistent cough, recognized it as a sign of tuberculosis because their symptoms had persisted for more than three weeks, even after undergoing some treatment at health facilities that proved ineffective.

**Table 6:** Participants’ suspicions.

Answers	Number of respondents and frequency n/N (100%)	Quotes
Tuberculosis	21/60 (35%)	“I’ve always suspected tuberculosis, as I have an elderly woman with this disease, I suspected that maybe here those things (bacteria) have already passed on to me, and I was afraid that the children would also become infected” (Female Adult Patient 21).
Nothing	16/60 (27%)	“Yeah, [I had] no suspicion. I didn’t have any suspicion, just the pain he (the child) feels. The way he coughs, I can’t even like watching him doing that while I’m standing there.” (Male Caregiver 23).
Cough	8/60 (13%)	“Hah, I thought it was a cough and that maybe it would go away, but nothing does. Nothing, I didn’t think of other illnesses, my opinion was that it’s just a cough, but when you get to the hospital they always ask you, so maybe they’ll say that this illness comes from these illnesses that exist, you’ll think it’s a cough and it’ll pass” (Male Adult Patient 14).
Asthma	5/60 (8%)	“I thought it was asthma. [...] then, as I live with elder people, they told me that it could be asthma and said that we could give the traditional medicine. If you get the traditional medicine, maybe it will get better, because every time you go to the hospital and they give you syrup when it’s over [the child] start[s] coughing again” (Male Caregiver 17).
Complications of HIV	4/60(8%)	“Hum (yes), [I thought it was] the virus, because I have it. I’m positive [...] for this disease we have, AIDS” (Female Caregiver 34).
Malaria	2/60 (3%)	“I thought I might have malaria” (Female Caregiver 35)
Side effects of TARV	1/60 (8%)	“[…]I just thought it must be an illness, or maybe it’s the pills that aren’t suitable for the treatment I’m taking” (Female Adult Patient 10).
Fatigue	1/60 (8%)	“Ohhh, I didn’t imagine anything, I can’t lie [...], my illness is the coughing and the cold. When I make (made) the *lha lha lha* (coughing sound), I thought it was tiredness from working so hard without anyone to help me. I didn’t think it was an illness” (Female Adult Patient 07).
COVID-19	1/60 (8%)	“I suspected, for example, coronavirus, as coronavirus is a disease that is easy to transmit”(Female Caregiver 06)
Consumption of inappropriate thing	1/60 (8%)	“It started on Monday [...] last week. Then I became patient, thinking it was because I’d eaten something that my stomach was rejecting. I noticed that it didn’t go away, but got worse” (Male Adult Patient 10).

Despite these differing perceptions of their condition, the majority of respondents reported not having sought any healthcare other than conventional treatments before consulting a physician. Nonetheless, our findings indicate that some respondents, including caregivers of paediatric patients who were not a TB contact and PWH, tended to seek health care later than caregivers of paediatric patients TB contacts.

This delayed behavior appears to be influenced, in part, by their intention of waiting for the next scheduled doctor visits, either for ARV collection or routine child health care, hoping to address their symptoms during the same visit.

One participant exemplified this approach, stating, “*What led me to come to the hospital is the problem (...) for two months I’ve been coughing, so I’ve had a fever since the 31st, while I’ve been coughing. [...] I talked to my friends. So [I realized that] I have to go for a tuberculosis consultation, because you can say you don’t have tuberculosis while I (or you) do. [...] but, as today was my day to go to the doctor, [...] I took the opportunity to get here to treat [...] this disease*” (Male Adult Patient 09).

This tendency to delay seeking care until a pre-scheduled visit suggests the need for greater emphasis on timely health-seeking behaviors, especially in populations managing multiple health concerns.

Additionally, our findings revealed that some participants reported taking pills like antibiotics purchased from private dispensers based on neighbors’ recommendations, while others, particularly female caregivers, resorted to homemade remedies such as syrup and steam inhalation before seeking medical care. This behavior underscores how conventional health care was often a secondary option, influenced by the perceived severity of the condition. For instance, one participant shared their rationale for delaying medical care, explaining, “*Nothing, I haven’t taken any pills yet. It’s the first time I’ve been to the hospital because I feel the (chest) pain continuing. So I decided to go to the hospital. I take pills for HIV, but I’m not sick, it’s just [the virus] in my blood. [...] I’m not sick yet*” (Female Adult Patient 12).

Similarly, a female caregiver described her attempts to manage her child’s condition at home before seeking professional help: “*Before I came to the hospital, I used to find ways to get onions. I’d cut it up, add vinegar or lemon, [...] [and] a little sugar with a spoon [and] mix it. Even if I wasn’t [at home], he (the child) used to go and take it (syrup) where I left it. So I saw that it didn’t help at all. But I saw that it wouldn’t go away. [Then][...] I thought about bringing him to the hospital*” (Female Caregiver 28).

These accounts highlight how caregivers and PWH often delay seeking medical attention, turning to self-treatment until symptoms worsen, emphasizing the need for targeted health education on the importance of timely care.

## Discussion

As far as we know, this is the first study in Mozambique that explores the knowledge of TB and healthcare-seeking behaviors among people with HIV, although similar a study has been done on caregivers of paediatric patients with symptoms of TB [16]. Despite this, our study remains relevant for improving health policies aimed at these groups by highlighting behaviors that hinder efforts to reduce TB incidence and related deaths within this population.

Overall, our results reveal that unhealthy healthcare-seeking behaviors are prevalent, reflecting a limited awareness of TB among our participants, particularly among those with HIV who were experiencing TB symptoms. For example, while a persistent cough was the primary reason for seeking healthcare, most participants reported having this symptom for about four weeks before undergoing TB screening.

Several factors contributed to the delay in seeking proper care, according to the participants’ accounts. One reason for the delay was that some participants, especially those living with HIV and caregivers of paediatric patients who were non-TB contacts, preferred to wait for their next scheduled doctor’s visit, such as for antiretroviral therapy (ARVs) refills or routine child health checkups. They hoped their symptoms could be addressed during these appointments, rather than seeking care sooner [16]. The literature sheds light on participants’ behavior through the lens of time and effort management, which is supported by differential models like the community-based ART delivery model (known as GAAC) [39,40]. The GAAC model was designed to simplify care by reducing the need for frequent clinic visits, addressing barriers like long travel distances, high transportation costs, and limited transportation options [39,40]. Under this system, groups of patients take turns visiting the clinic every month to collect medications, undergo consultations, and have tests done, which means each person typically only visits the clinic every six months [39,40]. While this model helps manage the aforementioned logistical challenges, our results suggest that it may also contribute to delays in accessing timely care, which could be detrimental when seeking prompt assistance for conditions like TB. These findings suggest that raising awareness about TB and its health implications among these two groups, particularly people living with HIV (PWH), is crucial. Additionally, improving the follow-up of TB symptoms, ensuring better attention from healthcare professionals, as well as enhancing support and surveillance within each patient group, are essential steps to effectively address the issue.

As well-documented in the literature [10,16,41–43], our results highlight that there continues to be a misinterpretation of symptoms, both by the participants themselves and potentially by the health professionals who assisted them before undergoing TB screening. This misinterpretation may have contributed to delays in seeking appropriate healthcare [16,43]. The data show that, despite cough being the most cited symptom of TB, the majority of participants either attributed their symptoms to no illness or other conditions, such as a normal cough (associated with cold or flu), asthma, HIV complications, malaria, side effects of ART, fatigue, or COVID-19. Only about a third of participants linked their symptoms to TB, and they did so only after experiencing prolonged symptoms and seeking either self-care or medical attention. These findings are consistent with a study conducted in Tanzania, which reported that caregivers experienced multiple delays in obtaining a TB diagnosis for their children due to repeated hospital visits and the deterioration of the child’s health before a TB diagnosis was finally made [42]. Similar studies have suggested that such delays could be attributed to the misinterpretation of symptoms, even by health professionals [10,16,41]. One study highlighted that multiple visits to healthcare facilities are a clear indication that the healthcare system is failing to diagnose TB effectively. In such cases, healthcare workers should be able to suspect TB in any patient with a chronic cough and a history of repeated visits to health facilities [10]. Research points to two reasons behind this misinterpretation, first limited knowledge among healthcare workers (HCWs) regarding TB patient profile, diagnosis and treatment, highlighting the challenges of training and keeping HCWs aligned with National TB Programme (NTP) guidelines in resource-limited settings [17]; and second, the often present non-specific signs, and TB symptoms, especially among PWH and children, whose symptoms tend to be sub-acute and insidious [12,16,41]. As a result, the TB symptoms are easily perceived as ‘non-alarming’ afflictions that do not prompt immediate (health)care-seeking [12,16,41].

We infer that this perception may explain why 20% of our participants reported that, influenced by themselves or by relatives and friends, initially self-medicated with pharmaceutical drugs or natural homemade remedies, leading to a delay in diagnosis [41,44].

Although our participants did not attribute their delays to seeking traditional healers, and mostly sought medical care, unlike a similar study conducted in the same setting [16], our results also indicate a lack of knowledge regarding TB among them. While 50% correctly identified airborne transmission, the other 50% demonstrated either a lack of awareness about TB transmission or associated it with sociocultural beliefs. This was particularly evident among people living with HIV (60%), who were more likely to hold such a misconception. These misconceptions included ideas such as TB being transmitted through touching an object of or engaging in sexual activity with someone whose family had not undergone purification after a relative’s death, or with a woman who had an abortion. Such misconceptions about TB have been identified in studies conducted both in Mozambique and other African countries as one of the risk factors contributing to delay in healthcare-seeking, often leading patients to turn to traditional healers [9,16,45–47]. As documented in some studies, these local beliefs about TB reflect the local cosmology in which illness or health conditions are seen as the consequences of one’s interactions with ancestors [16,48]. In this context, illness is not viewed as a natural or biological condition, but as a supernatural one, often reflecting the absence of a spiritual (ancestor) protection against witchcraft and the evil eye [16,48]. Therefore, cultural beliefs and myths surrounding TB influence whether or not people seek formal treatment and they respond to it [13,49], encourage people to consult traditional healers in times of distress and ill-health [9], or delay health-seeking [10,16]. Although our study did not find evidence linking cultural beliefs to participants’ willingness to seek care from traditional healers, the contrast with findings from a similar study conducted in the same setting [16] suggests that further studies are needed to explore potential changes in community knowledge, attitudes and practices related to TB.

Another limitation that emerged from our participants’ accounts was related to TB prevention. For example, most participants (34%) either reported not knowing how to prevent TB or cited avoiding the sharing of utensils, such as cups and plates, as the main preventive measures [16,50,51]. Although our findings show no difference between those aged 55–59 years and those aged 45–49 years, we corroborate with a Ghanaian study that found age negatively influenced TB myths and misconceptions. The study revealed that older participants (55–59 years) were more likely to believe TB could be transmitted through touch, sexual contact, sharing utensils, mosquito bites, or food [51]. It suggests that this may be due to their adolescence in the 1960s and 70s, when TB myths and misconceptions were widespread, compared to younger generations who likely had more exposure to TB education [51]. Two additional factors may explain this: the social construction of TB as a highly contagious disease, especially within the medical context, and the widespread practice of isolating TB patients as a preventive measure [52]. This approach likely contributed to the belief that TB was easily transmitted, leading to misunderstanding of the isolation measures and thus behaviors such as avoiding contact with the TB patients’ belongings or the utensils used by them, as illustrated by an adult female’ accounts: “*So, from today we will start living differently, [I will tell my son] you will not touch anything I will use”*.

These results suggest that in contexts of limited knowledge about TB, like this, health education should be directed at both individuals and communities and should not only impart knowledge, but also address the myths surrounding TB and discouragement of self-medication among these populations, particularly. Furthermore, combined interventions between different stakeholders, such as health sectors and patients’ entry-points, are needed to improve the follow-up of patients with TB symptoms and thus the active TB case finding. Although this study has helped to understand the caregivers of paediatric presumptive TB patients and PWH’s “knowledge and attitudes toward TB”, it has some limitations regarding the setting and the sample size. For example, the fact that the study was carried out in a rural area of southern Mozambique and that the sample of caregivers and adult patients did not follow the statistical criteria for determining representativeness does not lead us to generalize the results to other urban and rural areas. Additionally, during the process of translation, the removal of speech elements such as hesitations, repetitions, and incomplete sentences, though beneficial for readability, may have resulted in the loss of important cues related to uncertainty, emphasis, or emotional expression. Furthermore, the two-stage translation, from *Xichangana* to Portuguese and then to English, may have introduced an additional layer and may have also introduced additional opportunities for small errors or shifts in meaning, potentially affecting the accuracy and depth of the original statements. Lastly, due to logistical constraints, the study team was unable to turn to patients to verify the findings to ensure the validity of the data.

## Conclusions

This study explored TB knowledge and healthcare-seeking behaviors among PWH and caregivers of paediatric patients. Our results revealed that risky healthcare-seeking behaviors, such as delays in seeking care, were prevalent among participants experiencing TB symptoms, indicating limited awareness of the disease. Our findings suggest that factors such as the tendency to wait for the next scheduled doctor’s visit, driven possibly by a desire to minimize effort and cost, along with the misinterpretation of symptoms and misconceptions about TB, contributed to these delays in seeking appropriate healthcare. Therefore, raising awareness about TB and addressing the myths surrounding TB through health education, as well as improving the follow-up of TB symptoms through a combined approach across different patients’ entry points, is crucial.

## Supporting information

S1 Checklist Human participants research. (PDF)

S1 List List of Stool4TB Global Partnership collaborators. (PDF)

S1 Text In-depth interview guide. (PDF)

## Abbreviations

ART: antiretroviral therapy
CISM: Centro de Investigação em Saúde da Manhiça
COVID-19: coronavirus disease
HIV: human immunodeficiency vírus
PWH: people with HIV
SBS: socio-behavioral Science
TB: tuberculosis
WHO: World Health Organization
