# Supplementary material for: Gaps in TB-related knowledge and practices: An assessment of health care seeking behavior among adults with HIV and caregivers of paediatric patients with presumptive TB symptoms in Manhiça district, southern Mozambique
Source: PLOS Glob Public Health. 2025 Aug 18;5(8):e0004734. doi: 10.1371/journal.pgph.0004734 (PMC12360651; doi:10.1371/journal.pgph.0004734)
Supplement: S1 List — (PDF) [file pgph.0004734.s002.pdf]

List of Stool4TB Global Partnership Collaborators

Benilde Violeta Mudumane[1], Edson Mambuque[1], Farida Cebola[1], Jorcelina Rungo[1], Alberto Bila Junior[1], Neide Gomes[1], Shilzia Munguambe[1], Lee Joao Fonseca[1], Justina Bramugy[1], Katia Magul[1],Makhosazana Dlamini[2], Gcinile Dlamini[2], Nomathemba Dlamini[2], Nkulungwane Mthethwa[2], Babongile Nkala[2], Clement Gascua[2], Durbbin Mulengwa[2], Nokwanda Kota[2], Maia Madison[2], Busizwe Sibandze[2], Nontobeko Maphalala[2], Nosisa Shiba[2], Mbongeni Dude[2], Mangaliso Ziyane[2], Maria Nassolo[3], George Kasule[5], Moorine Sekkadde[3], Eric Wobudeya [3], Patricia Mwachan[3], Olivier Gillet[4], Elisa López-Varela[4], Belén Saavedra-Cervera[4], Lucía Caratalá-Castro[4], Sergi Sanz[4], Carlos Fernandez[4], Sujan Katuwal[5], Matthew Ang[5], Abigail Seeger[7], Anca Vasiliu[7], Andrew DiNardo[7], Rojelio Mejía[7], Jason Bacha[7], Debrah Vambe[7], Collins Musia[8], Irina Kontsevaya[8], Faith Dlamini[12], Fortunate Shabalala[12], Sindisiwe Dlamini[13], Gugu Maphalala[13], Lindiwe Dlamini[14], Sisi Dude[14], Mtafya Bariki[15], Nyanda Elias Ntinginya[15], Lilian Komba[16], Lwijisyo Minga[16], Lumumba Mwita[16].

[1]Centro de Investigação em Saúde da Manhiça (CISM), Manhiça, Mozambique

[2]College of Medicine and Texas Children's Hospital, Baylor, Eswatini

[3]Department of Medical Microbiology and Makerere University Lung Institute, Makerere University, Kampala, Uganda

[4]Institute for Global Health (ISGlobal), Barcelona, Spain

[5] Amsterdam UMC, location University of Amsterdam, Department of Global Health, Amsterdam Institute for Global Health and Development, Amsterdam, Netherlands

[7]Baylor College of Medicine and Texas Children's Hospital. Houston, Texas, United State of America

[8]Research Center Borstel, Leibniz Lung Center, Borstel, Germany

[12]University of Eswatini, Kwaluseni, Eswatini.

[13]Eswatini Health Laboratory Services, Mbabane, Eswatini.

[14]National TB Control Programme (NTCP), Ministry of Health, Mbabane, Eswatini

[15]National Institute for Medical Research-Mbeya Medical Research Centre, Mbeya, Tanzania.

[16]Baylor College of medicine Children's Foundation, Dar-Es-Salaam, Tanzania
