# Supplementary material for: Gaps in TB-related knowledge and practices: An assessment of health care seeking behavior among adults with HIV and caregivers of paediatric patients with presumptive TB symptoms in Manhiça district, southern Mozambique
Source: PLOS Glob Public Health. 2025 Aug 18;5(8):e0004734. doi: 10.1371/journal.pgph.0004734 (PMC12360651; doi:10.1371/journal.pgph.0004734)
Supplement: S1 Text — (PDF) [file pgph.0004734.s003.pdf]

Participants' perceptions and opinions about their presumptive TB condition

1.1. What made you come to this facility?

Explore:

- How long have you been in this state?
- Before you came to facility, what did you think it was? Why?
- During this time, were you submitted to any treatment to relieve the symptoms?
- If yes, which and by suggestion/referral of whom?
- When you were to facility, have you suspected any disease? Which it was and why?

1.2. When you were referred to the National Tuberculosis Program (NTP) office, what came in your mind first? Why?

Explore:

- Did you had any idea about what would happen to you there?
- How did you feel?

1.3. Which screening/exam were you submitted to?

1.4. When the screening was requested (*use the own term/word used by patient to describe the exam*), what did you think?

Explore:

- Could you tell us, what you felt? Why?
- At this time, what did you know about the disease (TB)?

  Explore:

  - In your opinion, how is TB transmitted?
  - Which are its symptoms?
  - How is TB avoided/prevented TB?

1.5. How do you imagine your life, in case of a positive result? Why?

Explore:

- What will change in your life and family? Why?
- How will your relationship be with your friends, family and people from your community?
